# Hydrothermal Synthesis of ZnO Structures Formed by High-Aspect-Ratio Nanowires for Acetone Detection

**DOI:** 10.1186/s11671-016-1563-x

**Published:** 2016-07-26

**Authors:** Zhen Cao, Yong Wang, Zhanguo Li, Naisen Yu

**Affiliations:** 1 College of Optoelectronic Engineering, Changchun University of Science and Technology, Changchun, 130022 China; 2 National Key Laboratory on High Power Semiconductor Lasers, Changchun University of Science and Technology, Changchun, 130022 China; 3 Liaoning Key Laboratory of Optoelectronic Films and Materials, School of Physics and Materials Engineering, Dalian Nationalities University, Dalian, 116600 China

**Keywords:** ZnO, Snowflake-like structures, Acetone Detection

## Abstract

Snowflake-like ZnO structures originating from self-assembled nanowires were prepared by a low-temperature aqueous solution method. The as-grown hierarchical ZnO structures were investigated by X-ray diffraction (XRD) and field-emission scanning electron microscopy (FESEM). The results showed that the snowflake-like ZnO structures were composed of high-aspect-ratio nanowires. Furthermore, gas-sensing properties to various testing gases of 10 and 50 ppm were measured, which confirms that the ZnO structures were of good selectivity and response to acetone and could serve for acetone sensor to detect low-concentration acetone.

## Background

As wide band gap materials, ZnO has been widely investigated for its potential applications [1–9]. Especially, it can be used in toxic and combustible gas detectors [10–13]. For gas sensor applications, ZnO is one of the promising materials. Moreover, low-dimensional ZnO materials have a large surface-to-volume ratio, which can be used as a potential material on gas-sensing devices [14–20]. ZnO nanostructures with different morphologies have been synthesized through a wide range of preparation methods [21, 22]. Among these methods, the low-temperature route is a suitable choice because of its simplicity, reproducibility, and cost-effectiveness and is attracting considerable attention [23, 24].

Acetone is a commonly used chemical solvent, which has been regarded as an extracting regent in the industry. In addition, acetone is a very important marker for noninvasive diagnosis of diabetes in the human breath aspects [25]. Thus, it is of great significance to develop a new type of acetone gas sensor that can be used as a noninvasive detector. The use of low-dimensional structures is a key technological factor in the creation of new functional and sensing devices [26].

We proposed a low-temperature method to prepare snowflake-like ZnO structures in this paper. The structures and morphologies have been investigated by X-ray powder diffraction (XRD) and field-emission scanning electron microscopy (FESEM). Micro-Raman and absorption spectrum were also performed to investigate the optical properties of the structures. Meanwhile, a gas sensor was made basing on the snowflake-like ZnO structures, and its gas-sensing properties were investigated. Particularly, the prepared sensor exhibited good selectivity and response to acetone which makes it as a good candidate for detecting low-concentration acetone.

## Methods

The snowflake-like ZnO structures were grown in aqueous solutions at a low temperature. The typical procedure was to use zinc nitrate (Zn(NO_3_)_2_) and hexamethyltetramine (C_6_H_12_N_4_) mixed solutions with the addition of NaF. The typical reaction process was listed as follows: 0.05 M Zn(NO_3_)_2_ and 0.02 M NaF were dissolved in distilled water. Then, 0.05 M C_6_H_12_N_4_ was added slowly under stirring condition. Afterward, the mixture solutions were reacted at 90 °C for 5 h. After washing with distilled water and pure ethanol, the sample was dried at 60 °C. Then, the obtained ZnOHF intermediate was baked at 400 °C for 2 h. Finally, the as-grown ZnO samples were cleaned with deionized water and dried in the air.

The gas-sensing properties were performed by using a static state gas-sensing measurement system. As-prepared ZnO nanostructures were dispersed with deionized water to form a paste. Afterwards, it was coated onto a ceramic substrate. In addition, three pairs of gold interdigital electrodes were made to form a ZnO sensing film. The thickness of the ZnO sensing film was about 300 nm.

## Results and Discussion

**Fig. 1:**
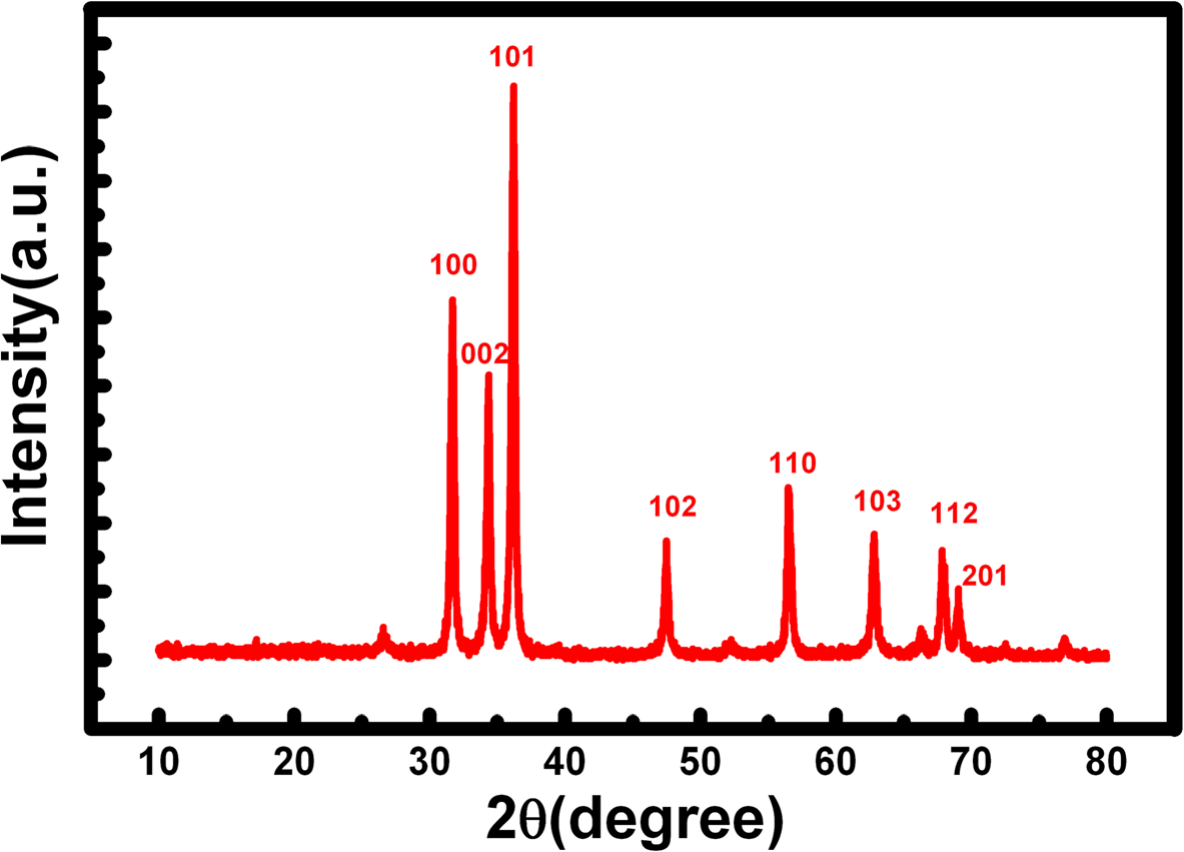
XRD patterns of the ZnO prepared by a hydrothermal method after the heat treatment

Figure 1 shows the XRD results of the ZnO samples. It can be clearly seen that all the diffraction peaks could be attributed to the ZnO wurtzite hexagonal phase. Meanwhile, no impurity peaks are detected, which indicates the high purity of the ZnO.

**Fig. 2:**
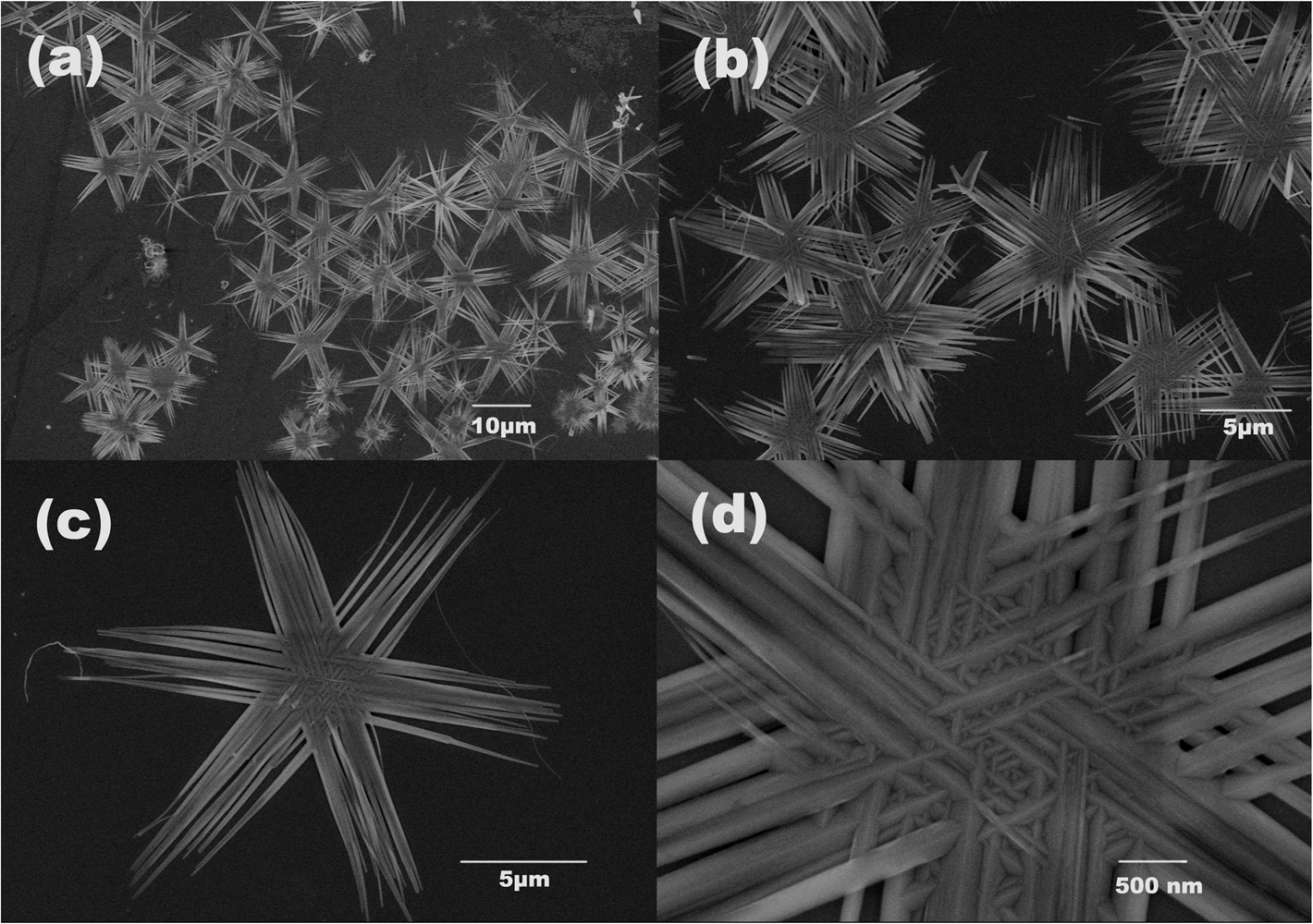
FESEM images of the as-synthesized ZnO nanostructures. **a** Panoramic view. **b** Enlarged part of the sample. **c** Individual ZnO snow-like structure. **d** Further magnification of a single ZnO snow-like structure

Figure 2a, b displays a low-magnified morphology of the sample over a large area. It can be clearly seen that the sample was consisted of monodisperse snowflake-like microstructures. The diameter is around 15 μm, and almost all of them have a uniform size and present one kind of structure. The further magnified pictures are shown in Fig. 2c, d; it can be seen that the nanowires are joining with each other, and a network structure has been formed by interconnected nanowires. The SEM images indicate the formation of snow-shaped hierarchical ZnO architecture. The hierarchical ZnO architecture is found to be made up of nanowires which were self-assembled to form snowflake-shaped assemblies. The synthesis of hexagonal ZnO nanostructures has been reported [27]. However, the approach based on the solution method to prepare this structure is rarely disclosed. The reason why the structures grow into hexagonal symmetry could be attributed to the hexagonal symmetry of the ZnO core. The ZnO core provides its six prismatic crystal planes facets, which serve as growth platforms for nanowire units. Moreover, the mechanism of forming snowflake-like ZnO structures can be attributed to NaF, and it can react with Zn(NO_3_)_2_ to form ZnOHF intermediate. Meanwhile, it can accelerate nanowires that are weaved into netlike structures that overlap with each other [28]. The reaction process of the ZnOHF intermediate could be proposed as follows:

1 $$ {\mathrm{Zn}}^{2+} + {\mathrm{F}}^{\hbox{-}}\to {\mathrm{Zn}\mathrm{F}}^{+} $$

2 $$ {\mathrm{NO}}^{3\hbox{-} } + {\mathrm{H}}_2\mathrm{O}+2{\mathrm{e}}^{\hbox{-}}\to {\mathrm{NO}}^2 + 2{\mathrm{OH}}^{\hbox{-} } $$

3 $$ {\mathrm{ZnF}}^{+} + {\mathrm{OH}}^{\hbox{-}}\to\ \mathrm{ZnOHF} $$

**Fig. 3:**
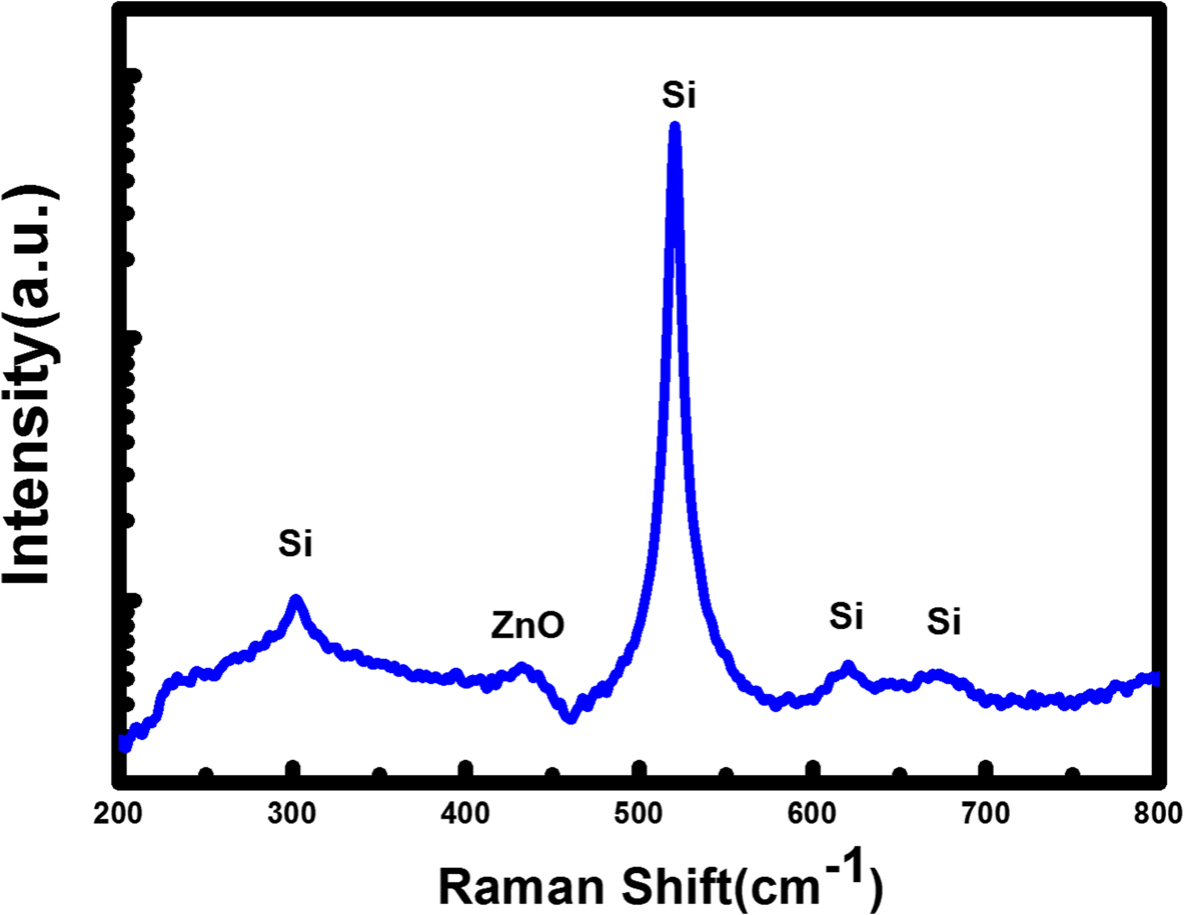
Micro-Raman spectrum of an as-grown individual ZnO structure

Figure 3 shows the micro-Raman spectrum of an as-grown individual ZnO structure which was dipped on Si. The peak located at 303, 620, and 670 cm^−1^ are Si vibration modes [29]. The peak with a weak intensity at 434 cm^−1^ can be attributed to E_2_(H) mode, which are the intrinsic characteristic of the Raman active mode of wurtzite hexagonal ZnO [30]. Meanwhile, it downshifts in comparison with the stress-free bulk ZnO value of 437 cm^−1^, indicating that it suffers from tensile stress.

**Fig. 4:**
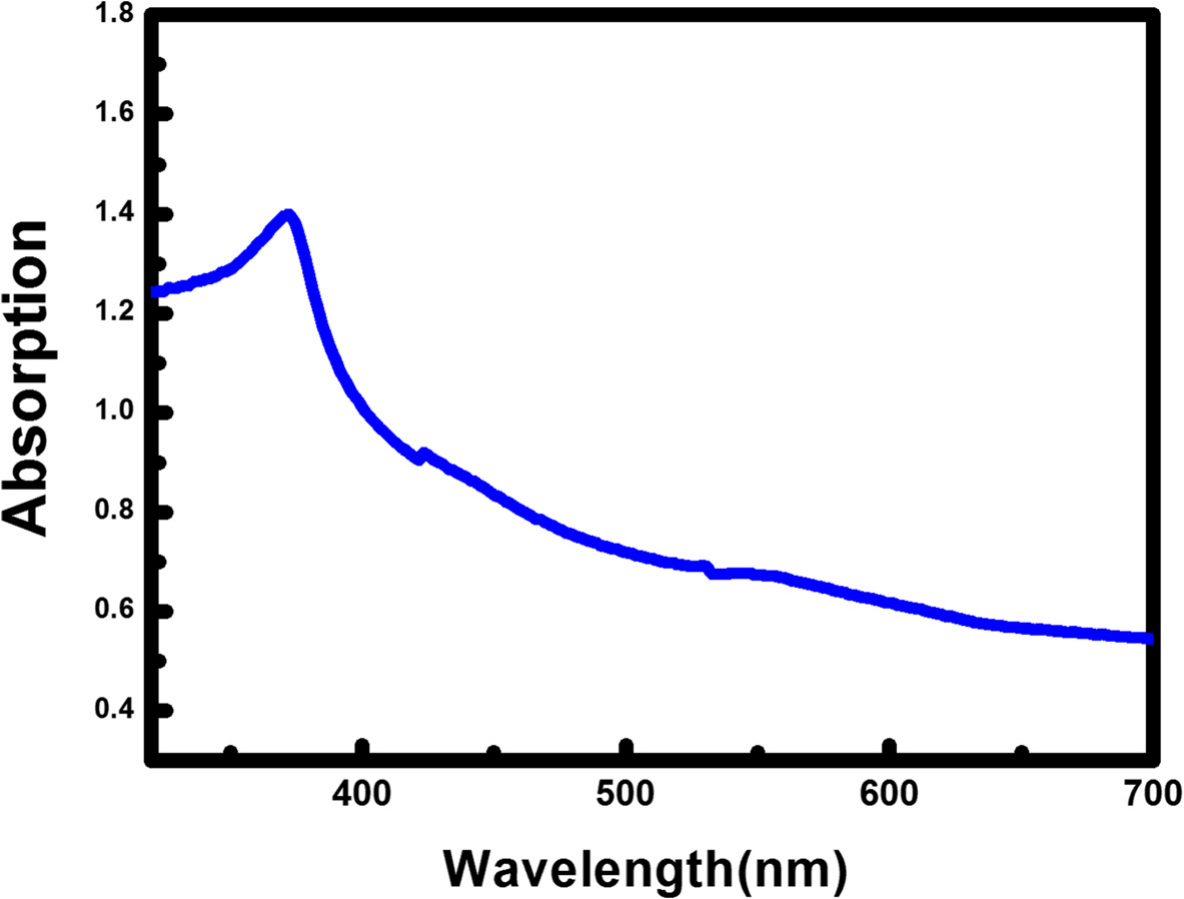
Absorption spectrum of an as-grown ZnO structure

Figure 4 displays the absorption spectrum of an as-grown ZnO structure. It shows a strong UV absorption and a weak visible light absorption. Meanwhile, the cut-off wavelength is around 377 nm, which shows a 3-nm blue shift in comparison with the ZnO bulk material (380 nm). This shift corresponds to the quantum confinement effects of nanostructures [31].

The gas-sensing properties were performed by using a static state gas-sensing measurement system [32]. The as-prepared ZnO nanostructures were coated onto a ceramic substrate (250~300 μm in thickness), dried at 100 °C for 2 h, and annealed at 500 °C for 2 h in air. Finally, a Ni-Cr heating wire was inserted into the ceramic tube to form an inside-heated gas sensor. Voltage division circuit was adopted to measure the export voltage on the sensor. Meanwhile, an external resistor (*R*_L_) was connected in series with the obtained sensor under the bias of 10 V. The gas response value (*S*) was defined as a ratio of the electrical resistance of the sensor in air (*R*_a_) to that in a testing gas (*R*_g_): *S* = *R*_a_/*R*_g_, and *R*_a_ = *R*_L_(10-*V*_air_)/*V*_air_, *R*_g_ = *R*_L_(10-*V*_gas_)/*V*_gas_, where *V*_air_ was the export voltage of *R*_L_ in air and *V*_gas_ was the voltage in the testing gas [32].

**Fig. 5:**
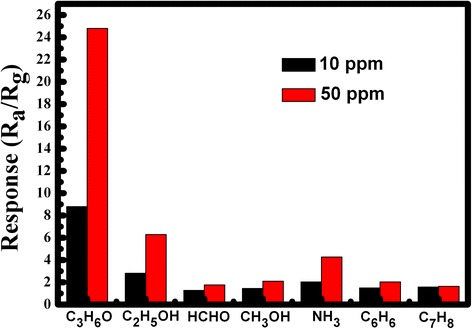
Response of ZnO nanostructures to 10 and 50 ppm various gases at 350 °C

The gas responses of ZnO nanostructures to various testing gases of 10 and 50 ppm are performed with an operating temperature of 350 °C. Figure 5 exhibits the histogram showing the response of ZnO sensor to various gas vapours, including acetone(C_3_H_6_O), ethanol (C_2_H_5_OH), formaldehyde (HCHO), methanol (CH_3_OH), ammonia (NH_3_), benzene (C_6_H_6_), and methylbenzene (C_7_H_8_). It can be clearly seen that acetone displayed remarkable higher responses in comparison to the other gases. Under the gas concentrations at 10 ppm, the highest response of the sensor is around 9 to acetone, and those of the other gases are no greater than 3. Moreover, the response to acetone is particularly stronger than the other gases under the gas concentrations at 50 ppm, the response magnitude was about 25, while the response to other gases was no more than 7. It is obvious that the sensor based on snow-like structures exhibits excellent acetone sensing.

**Fig. 6:**
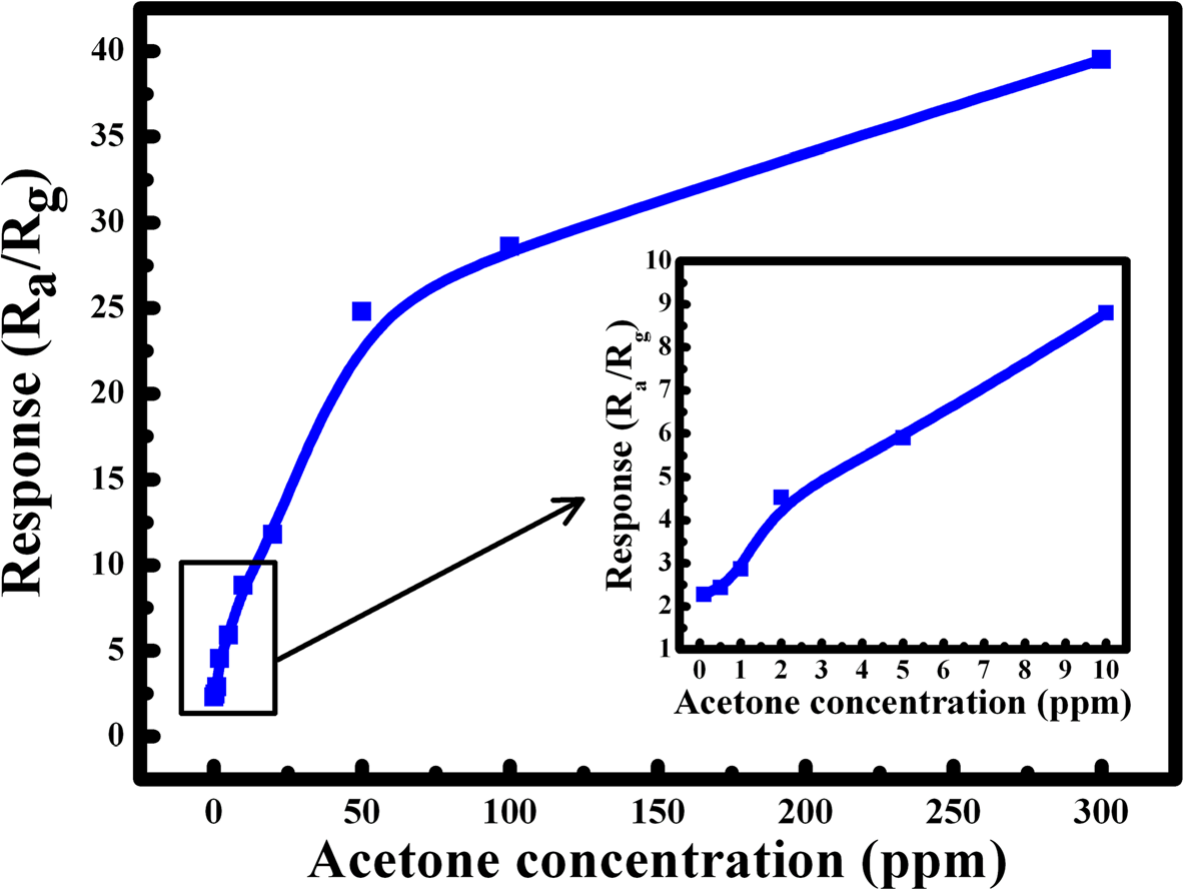
Response to different concentrations of acetone at an operating temperature of 350 °C

Figure 6 demonstrates the concentration-dependent sensitivity of the ZnO sensor based on snow-like structures for acetone detection with an operating temperature of 350 °C. It can be seen that the response below 10 ppm acetone increases quickly when the gas concentration is increasing. Above 10 ppm, the response slowly increases with increasing acetone gas concentration. Moreover, the inset in Fig. 6 shows the linear curve below 10 ppm. It indicates the ZnO nanostructures are much more suitable to detect acetone with a low concentration.

The working mechanism of ZnO gas sensors can be attributed to the conductivity changes. The reaction between the testing gas and the oxygen species located on the surface of the ZnO structures could cause resistance change. In our case, when the sensor based on snow-like structures is exposed to acetone gas, the acetone gas is oxidized by the oxygen species to form formaldehyde and then increase conductance. The response to acetone with a low concentration could be ascribed to the snow-like sensor structure, which has a much bigger specific surface area than other conventional sensing structures, which could provide a larger adsorption region then increase the amount of gas molecules adsorbed on the sample surface. Moreover, the formation of nanowire junctions may be another reason for the response enhancement [33]. The junctions are considered as the active sites, which can increase the gas response sensitivity.

## Conclusions

In summary, snowflake-like ZnO structures were synthesized by a simple low-temperature way. The structures are constructed of high-aspect-ratio ZnO nanowires. Moreover, the snowflake-like structure sensor exhibits excellent acetone sensing, which are much suitable to detect acetone with a low concentration.
